# Age and Sex Specific Trends in Incidence of Juvenile Idiopathic Arthritis in Danish Birth Cohorts from 1992 to 2002: A Nationwide Register Linkage Study

**DOI:** 10.3390/ijerph18168331

**Published:** 2021-08-06

**Authors:** Isabel Cardoso, Peder Frederiksen, Ina Olmer Specht, Mina Nicole Händel, Fanney Thorsteinsdottir, Berit Lilienthal Heitmann, Lars Erik Kristensen

**Affiliations:** 1 The Parker Institute, Bispebjerg and Frederiksberg Hospital, 2000 Frederiksberg, Denmark; isabel.dos.santos.cardoso@regionh.dk (I.C.); EEK@garp.dk (P.F.); Ina.Olmer.Specht@regionh.dk (I.O.S.); Mina.Nicole.Holmgaard.Handel@regionh.dk (M.N.H.); fanney.thorsteinsdottir@regionh.dk (F.T.); lars.erik.kristensen@regionh.dk (L.E.K.); 2 The Boden Institute of Obesity, Nutrition, Exercise & Eating Disorders, University of Sydney, Sydney, NSW 2006, Australia; 3 The Department of Public Health, Section for General Medicine, University of Copenhagen, 1014 Copenhagen, Denmark

**Keywords:** incidence, juvenile idiopathic arthritis, epidemiology, Denmark

## Abstract

This study reports age- and sex-specific incidence rates of juvenile idiopathic arthritis (JIA) in complete Danish birth cohorts from 1992 through 2002. Data were obtained from the Danish registries. All persons born in Denmark, from 1992–2002, were followed from birth and until either the date of first diagnosis recording, death, emigration, 16th birthday or administrative censoring (17 May 2017), whichever came first. The number of incident JIA cases and its incidence rate (per 100,000 person-years) were calculated within sex and age group for each of the birth cohorts. A multiplicative Poisson regression model was used to analyze the variation in the incidence rates by age and year of birth for boys and girls separately. The overall incidence of JIA was 24.1 (23.6–24.5) per 100,000 person-years. The rate per 100,000 person-years was higher among girls (29.9 (29.2–30.7)) than among boys (18.5 (18.0–19.1)). There were no evident peaks for any age group at diagnosis for boys but for girls two small peaks appeared at ages 0–5 years and 12–15 years. This study showed that the incidence rates of JIA in Denmark were higher for girls than for boys and remained stable over the observed period for both sexes.

## 1. Introduction

Juvenile idiopathic arthritis (JIA) is a disease that comprises a heterogeneous group of inflammatory arthritis phenotypes, which are characterized by arthritis of unknown origin in one or more joints that persists for more than 6 weeks and with onset before the age of 16 years [1,2]. JIA is the most common chronic rheumatoid disease in children [3]. Based on the International League of Associations for Rheumatology (ILAR) classification system, JIA encompasses seven disease sub-types (oligoarticular, polyarticular rheumatoid factor (RF)-negative, polyarticular RF-positive, systemic, enthesitis-related arthritis, psoriatic arthritis, and undifferentiated) that differ by the number of joints affected and other disease features [4,5]. Overall JIA is characterized by joint inflammation, but the course of the disease and its associated conditions depend on the subtype, severity of the disease and treatment given [6]. When the disease is not properly controlled, it may lead to joint destruction and physical disability [6,7]; and extra-articular manifestations, such as uveitis, which are not uncommon either [7]. This condition can greatly decrease quality of life, physical function and development [5], and thus impose a substantial burden for the affected children, their parents and society [1]. JIA’s etiology and pathogenesis remain unclear [4], but it is considered a multifactorial disease involving both genetic and environmental factors [8], in which immune dysregulation is a key pathogenic factor [3].

Quantifying the occurrence of JIA in populations can provide important information about the disease, and specifically the incidence rates can provide insightful information related to the etiology and dynamics of the disease over time. A systematic review of prevalence and incidence of JIA reported that incidence and prevalence estimates varied greatly among studies and countries [2]. Reported incidence rates ranged from 1.6 to 23 and prevalence ranged from 3.8 to 400/100,000; overall these estimates were higher for girls than boys [2]. Berntson et al. (2003) [9] conducted a study over an 18-month period to ascertain the incidence of JIA according to the ILAR and European League Against Rheumatism (EULAR) criteria, within defined areas in the Nordic countries, including Denmark. This study reported incidence rates (ILAR criteria) of 9 (range: 5–12) and 16 (range: 9–23)/100,000 children/year in two different areas in Denmark: east Denmark (which comprises the islands of Sjaelland, Bornholm, Møn and Lolland-Falster, but not the county of Copenhagen) and Aarhus, respectively [9].

In order to measure the true incidence, a population-based approach is necessary and, for uncommon diseases like juvenile idiopathic arthritis, incidence rates should be measured over a long period to gain good accuracy [9]. Furthermore, age- and sex-specific incident rates can be useful to better understand the nature and distribution of the disease. By taking advantage of the access to unique national administrative registers available in Denmark, this project aimed to ascertain age- and sex-specific incidence rates of JIA in all children born in Denmark from 1992 through 2002.

## 2. Materials and Methods

### 2.1. Data Sources and Variables

This registry-based study obtained data from the Danish health care registers. In Denmark, every citizen has a unique personal civil registration number (CPR) that is used in all national registries. Using the CPR number, it is possible to link individual-level information from nationwide Danish registries [10] and track individuals over time [11]. The Danish Civil Registration System is an administrative register that contains information from all persons residing in Denmark, including sex, date and country of birth, updated information on vital status and emigration along with the date of these events [10,11]. The Danish National Patient Register (DNPR) contains information about hospital contacts, including date, diagnoses (coded according to the International Classification of Diseases (ICD) eight revision (ICD-8) (before 1994) and tenth revision (ICD-10)) and associated procedures [12]. In this study, the DNPR was therefore used to identify individuals with JIA (JIA cases) and to retrieve related information, such as the date of diagnosis. 

### 2.2. Source Population 

For the purpose of determining the incidence rates of JIA in Denmark the population at risk was defined as all persons born in Denmark from 1 January 1992 to 31 December 2002, followed from birth and until either the date of first time of diagnosis recording of JIA, death, emigration from Denmark, 16th birthday or administrative censoring (17 May 2017), whichever came first.

### 2.3. Incident JIA

Incident cases of JIA were children aged <16 years, born in Denmark between 1 January 1992 to 31 December 2002, and recorded in the DNPR with a first time ICD code for JIA from 1992 until 17 May 2017. From 1 January 1995 all outpatient and emergency room contacts were mandatorily included in DNPR. However, since the vast majority of JIA cases were already registered at public hospital records, due to the presence of specialized hospital departments, we do not expect this change in diagnosis procedure to have influenced our results. 

ICD codes for JIA were defined as: (1) JIA’s ICD codes (ICD-8: 712.09 and ICD-10: M08.0, M08.0A, M08.0B, M08.1, M08.2, M08.2A, M08.2B, M08.3, M08.4, M08.8, M08.8A, M08.9); and (2) RA’s ICD codes when diagnosis was made before age of 16 years (ICD-8: 712.19, 712.29, 712.39 and ICD-10: M06.0, M06.8, M06.9, M12.3). The date of onset was defined as the first time the ICD code was registered in the DNPR.

### 2.4. Statistical Analysis

In each of the Danish birth cohorts from 1992 until 2002, the number of incident JIA cases was classified by sex and age group (ages 0 to 15 years, divided into 1-year age groups). Age- and sex-specific incidence rates were calculated for each birth cohort, where the number of incident JIA cases in each stratum was divided by the total person-years at risk in that same stratum. The total person-year risk was calculated by summing up the time that each person remained under observation and was at risk of becoming a case. 

A multiplicative Poisson regression model, with log person-years as offset, was used to analyze the variation in the incidence rates by age and year of birth for boys and girls separately. The birth cohort 1997, which was the middle birth cohort, was used as reference for the incidence rate ratio calculation.

R version 3.5.1 [13] was used for statistical analysis.

### 2.5. Ethical Considerations

This is a registry-based study and according to Danish law, use of data does not require approval from an ethical committee. Permission to access and link the registries using the CPR requires permission from the Danish Data Protection Agency, which has been granted (j.nr: 2012-58-0004, BFH-2017-124). This study was conducted in accordance with the Strengthening the Reporting of Observational Studies in Epidemiology guidelines [14].

## 3. Results

Table 1 shows the characteristics of the source population and individuals diagnosed with first time JIA, by year of birth (frequency of particular types of JIA is available in Table S1). From 1992 through 2002 there were a total of 738,145 births; and during the study period, a total of 2770 individuals were diagnosed with a first diagnosis of JIA before their 16th birthday. There were approximately 67,000 births per year, with equal sex distribution. Among those diagnosed with JIA, the incidence rate was higher for girls (29.9 (29.2–30.7) per 100,000 person-years) than for boys (18.5 (18.0–19.1) per 100,000 person-years). The incidence rate ranged from 26.0 (23.9–28.4) to 36.3 (33.6–39.1) per 100,000 person-years for girls born in 1993 and 2001, respectively; and from 15.0 (13.5–16.8) to 21.6 (19.7–23.8) per 100,000 person-years for boys born in 1994 and 2001 (Table 1 and Figure S1). 

Birth cohort-specific incidence rates of JIA by age group at diagnosis fluctuated considerably within each cohort, for both boys and girls; and overall were smoother for girls than for boys (Figures S2 and S3, respectively). 

**Figure 1:**
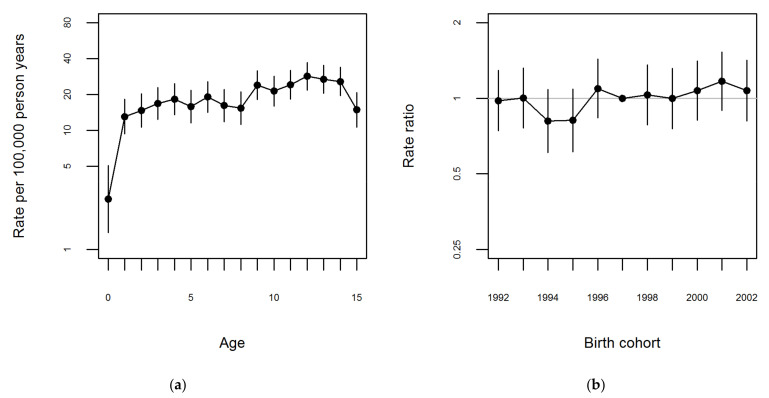
Estimates from the age-cohort model fitted to data on first time JIA diagnosis, for boys. Legend: (**a**) Age specific rates per 100,000 person-years and 95% CI for boys born in 1997. (**b**) Rate ratio relative to 1997 birth cohort for boys.

**Figure 2:**
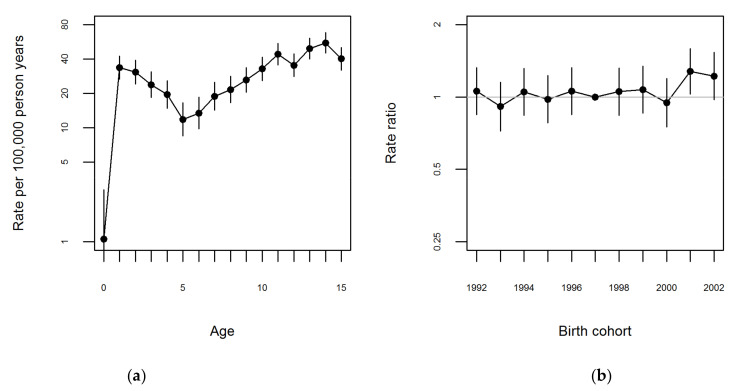
Estimates from the age-cohort model fitted to data on first time JIA diagnosis, for girls. Legend: (**a**) Age specific rates per 100,000 person-years and 95% CI for girls born in 1997. (**b**) Rate ratio relative to 1997 birth cohort for girls.

A multiplicative Poisson regression model with log person-years as offset was used to analyze the variation in incidence rates across age and year of birth for boys and girls separately. Figure 1a and Figure 2a show the estimated incidence rates for 1997 for boys and girls, respectively.

There were no evident peaks for any age group at diagnosis for boys (Figure 1a and Figures S3 and S4). For girls two peaks were visible, however not markedly; with a first peak between the ages 0–5 years and the second between ages 12–15 years (Figure 2a and Figures S2 and S4). Figure 1b and Figure 2b show the rate ratios relative to the 1997 birth cohort for boys and girls, respectively. Overall, the rate ratios (Figure 1b and Figure 2b) as well as the sex-specific incidence rates (Figure S1) were very stable across birth cohort years.

## 4. Discussion

In this nationwide study we observed that the incidence rates of JIA were higher for girls than for boys and remained stable during the study period for Danish birth cohorts from 1992 through 2002. In addition, there were no evident peaks observed for any of the age groups for boys, while two peaks were visible for girls.

In our study, the total incidence rate of JIA for both sexes was 24.1 (23.6–24.5) per 100,000 person-years, which is close to, but slightly higher than the incidence rates found in Denmark in two other studies, namely Berntson et al. [9] and Ostergard et al. [15]. Berntson et al. reported incidence rates of 9 (range: 5–12)/100,000/year and 16 (range: 9–23)/100,000/year in 2 different areas of Denmark over a period of 18 months starting on 1 July 1997 [9]; and Ostergaard et al. conducted a retrospective study in the County of North Jutland during the periods of 1970–1977 and 1978–1986, in which cases were retrieved from general pediatric clinics, and reported an incidence rate of 6–8 cases per 100,000/years [9,15]. Differences in study design, cases ascertained methods and/or the JIA classification criteria in use can eventually justify the differences observed between our study and these two studies conducted in Denmark [9,15]. Our study results were nationwide population based whereas the previous Danish studies were from selected areas in Denmark, only [9,15]. Furthermore, the register-based data used in our study were systematically collected, with no obvious selection bias as JIA is treated in hospitals in Denmark. Therefore, we expect that we have captured all registered cases of JIA. However, we cannot rule out the risk of misclassification regarding diagnosis code. In addition, the rate found in our study is in agreement with the rates found by Berntson et al. in other Nordic countries (over a period of 18 months starting on 1 July 1997), namely in Norway (19 (range: 7–31) and 23 (range: 10–36)/100,000/year from 2 different regions) and Finland (21/100,000/year (range: 15–26) in the Helsinki region) [9], but higher than the rates found in Sweden (15/100,000/year (range: 12–18)) and Iceland (7/100,000/year (range: 1–13)) [9]. The incidence rate reported in our study is one of the highest, which is in agreement with the highest rates reported in a systematic review published in 2014 [2]. This review [2] included a total of 33 studies on incidence of JIA published between 1983 (Towner et al., USA 1960–1980) [16] and 2010 (Modesto et al., Spain 2004–2006) [17], with most of them being from Europe and North America; and reported an overall annual incidence rate of JIA that ranged from 1.6/100,000/year in France (1982) [2,18] to 23/100,000/year in Finland (2000) [2,19]. The highest rates have thus been reported in the Nordic European countries [9,19,20,21], and among them, Sweden [9,22,23] and Iceland [9] have the lowest rates. The lowest rates in Europe have been found in France [18,24], Germany [25,26] and Spain [17,27]. One could argue, however, that these countries do not have complete nationwide registries collecting in- and out-hospital diagnosis coding and thus might not catch all available cases.

The female predominance observed in our study is in agreement with findings from several previous studies [16,17,19,21,23,27]. In addition, among the girls but not the boys we observed a bimodal distribution of the age at diagnosis, which was used as a proxy for age of onset. The first peak was visible between the ages 0–5 years and the second between ages 12–15 years. This observation is in agreement with a Swedish study (from 1 January 1984 to 31 December 1988) that reported one peak in children younger than 5 years and the other peak among 10–15 years old [23]. A Spanish study (Modesto et al., Spain 2004–2006) observed a trend to a bimodal distribution for both girls and boys together, with higher incidence in the group of 1–6 years old and 12–15 years old [17]. Berntson et al. [9] and Sullivan et al. (Michigan, from 1961 to 1973) [28], both reported an early peak of age at onset in girls around/at 1–3 years old; and an American study (in years 1960–1979) reported one peak for girls at 0–4 years [16]. In our study, we did not identify any peaks for boys, which is in agreement with the study from Berntson et al. [9] in the Nordic countries; but others did [16,28]. One reason why we did not identify a bimodal distribution among boys, could be that axial spondyloarthritis, which is driving the second peak in the bimodal prevalence, is most often managed in specialist care in Denmark and thus may not have been caught by the registries.

In our study the incidence rates remained stable across birth cohorts for both boys and girls; however other studies have reported different results. Peterson et al. [29] reported that the incidence of JIA in Minnesota had decreased in the last decade of the study period, in 1960–1993. Later on, Krause et al. [30] evaluated the incidence of JIA in Minnesota in 1994–2013. Together with the data from Peterson et al. [29], Krause et al. [30] were able to evaluate its trends in the period 1960–2013. Krause et al. reported that the overall incidence did not change significantly in the period of 1960–2013 [30]. The authors also performed a sub-analysis by sex and observed that the incidence declined among girls, but did not change significantly among boys [30]. In contrast, Kaipiainen-Seppänen and Savolainen reported that the incidence of JIA in Finland in 1995 was significantly higher than the incidence found in previous years (1980, 1985 and 1990) [21].

Overall, the reported incidence rates of JIA vary considerably between studies, which could be due to e.g., the study design, differences in cases ascertainment methods, the classification system used and the geographical area of study [2]. It can thus be difficult to compare incidence rates and understand if those are true differences or if they are due to the aforementioned methodology diversity among studies [2].

### Strengths and Limitations

One of the strengths of this study is the comprehensive nationwide, population-based data from the mandatory Danish registration system, which allowed the almost complete follow-up of entire Danish birth-cohorts throughout a long-period of time in a country with a universal health care system; making this study virtually unaffected by referral and selection bias [31]. The Danish administrative and health registers can and are often used for epidemiological research and, in general, they are characterized by good quality and validity [11,31]. However, a few limitations should be noted. The DNPR, at its onset in 1977, recorded only information on all inpatients’ contacts from somatic wards. Outpatients’ contact with emergency departments have been mandatorily registered from 1 January 1995 and onwards (with a voluntary trial registration in 1994), thus the registry was not complete prior to 1995. From 1995 onwards, the DNPR covers data from all inpatients (both somatic and psychiatric inpatients), from psychiatric and somatic outpatients and emergency departments. Private hospitals and private outpatient specialty clinics were not obligated to report to the DNPR before 2003 [12,31]. We do not expect this to influence the results markedly, as the vast majority of JIA cases are registered at public hospitals, due to the presence of specialized hospital departments. Thus, one should expect that the majority of the JIA cases were diagnosed by a specialist and that the registration of the disease is accurate. However, no studies of validity and completeness of JIA diagnoses recorded in the DNPR have been identified to confirm that. In our study, case definition was based on ICD-8 and -10 codes, which should reflect the classification system in use at the time of the diagnose registration. Until 1997, there were two classification criteria in use, namely the American College for Rheumatology (ACR) criteria and the EULAR criteria. In 1997, a new classification was developed (ILAR), which was revised in 2001. In 1996, a study reviewed literature published between 1983 and 1995, when ACR and EULAR were widely used, with the objective to analyze the factors that could explain the differences in the reported frequency of chronic arthritis in childhood [32]. It was reported that the incidence rates and prevalence were not affected by the use of different diagnostic criteria [32,33]. Thus, even though the detailed information on classification was not available in our data, we find it unlikely that the different classification criteria affected our results.

## 5. Conclusions

This is the first nationwide population-based study describing the incidence of JIA in Denmark. The incidence rates were higher for girls than for boys and remained stable across the observed period for both sexes.

## Figures and Tables

**Table 1:** Characteristics of the source population and individuals diagnosed with first-time juvenile idiopathic arthritis (JIA).

	Total Population Born between 1992–2002			Population Diagnosed with JIA					
Year of Birth	Person-Years			Incidence Rate			Number of Cases		
	Total	Boys	Girls	Total	Boys	Girls	Total	Boys	Girls
1992	1,064,050	546,621	517,429	24.0 (22.5–25.5)	18.1 (16.4–20.0)	30.1(27.8–32.7)	255	99	156
1993	1,058,490	543,585	514,905	22.2 (20.8–23.7)	18.6 (16.8–20.5)	26.0 (23.9–28.4)	235	101	134
1994	1,093,464	559,228	534,236	22.3 (20.9–23.8)	15.0 (13.5–16.8)	29.9 (27.7–32.4)	244	84	160
1995	1,092,764	562,261	530,503	21.3 (20.0–22.8)	15.1 (13.6–16.8)	27.9 (25.7–30.3)	233	85	148
1996	1,058,027	544,222	513,805	25.0 (23.6–26.6)	20.2 (18.4–22.2)	30.2 (27.8–32.7)	265	110	155
1997	1,061,675	545,903	515,772	23.4 (21.9–24.9)	18.5 (16.7–20.4)	28.5 (26.2–31.0)	248	101	147
1998	1,037,950	534,459	503,491	24.4 (22.9–26.0)	19.1 (17.3–21.1)	30.0 (27.6–32.5)	253	102	151
1999	1,038,340	531,331	507,009	24.4 (22.9–25.9)	18.4 (16.7–20.4)	30.6 (28.2–33.1)	253	98	155
2000	1,051,363	539,234	512,129	23.3 (21.9–24.8)	19.8 (18.0–21.9)	26.9 (24.7–29.3)	245	107	138
2001	1,011,038	517,397	493,641	28.8 (27.1–30.5)	21.6 (19.7–23.8)	36.3 (33.6–39.1)	291	112	179
2002	933,984	480,327	453,657	26.6 (24.9–28.3)	20.0 (18.0–22.1)	33.5 (30.9–36.3)	248	96	152
Total	11,501,144	5,904,567	5,596,578	24.1 (23.6–24.5)	18.5 (18.0–19.1)	29.9 (29.2–30.7)	2770	1095	1675

## Data Availability

Not applicable.

## Supplementary Materials

The following are available online at https://www.mdpi.com/article/10.3390/ijerph18168331/s1, Table S1: Frequency of types of JIA. Figure S1: Sex-specific incidence rates per 100,000 PY of JIA in Danish birth cohorts from 1992–2002, Figure S2: Birth-cohort specific incidence rates per 100,000 PY of JIA by age group for boys, Figure S3: Birth-cohort specific incidence rates per 100,000 PY of JIA by age group for girls, Figure S4: Total incidence rate of JIA by age group for boys and girls.
