# Supplementary material for: Age and Sex Specific Trends in Incidence of Juvenile Idiopathic Arthritis in Danish Birth Cohorts from 1992 to 2002: A Nationwide Register Linkage Study
Source: Int J Environ Res Public Health. 2021 Aug 6;18(16):8331. doi: 10.3390/ijerph18168331 (PMC8394352; doi:10.3390/ijerph18168331)
Supplement: Supplementary file 1 [file ijerph-18-08331-s001.zip › ijerph-1255490-supplementary.pdf]

Table S1. Frequency of types of JIA.

	ICD codes for JIA	%
ICD-8	71,209	0.11
ICD-10*	M08.0	27.40
	M08.0A	1.26
	M08.0B	1.30
	M08.1	0.58
	M08.2	4.04
	M08.2A	0.43
	M08.2B	0.07
	M08.3	6.68
	M08.4	24.56
	M08.8	1.62
	M08.8A	2.96
	M08.9	23.97
	**ICD codes for RA when diagnosis was made before age of 16 years**	**%**
ICD-8	71,219	0
	71,229	0.04
	71,239	0
ICD-10*	M06.0	0.72
	M06.8	0.22
	M06.9	3.00
	M12.3	0.04

"M" indicates that the diagnosis relates to "Musculoskeletal and connective tissue disease". "M" in conjunction with the numbers "0" and "8", for example, indicates that the diagnose relates to the category of "Juvenile arthritis". M08.0: Juvenile rheumatoid arthritis. M08.0A: Juvenile rheumatoid arthritis with rheumatoid factor. M080B: Juvenile rheumatoid arthritis without rheumatoid factor. M08.2: Juvenile arthritis with extra-articular manifestations. M08.2A: Still's disease with onset before turning 16 years old. M08.2B: Still's disease unspecified. M08.8: Other juvenile arthritis. M08.8A: Enthesitis-related arthritis.

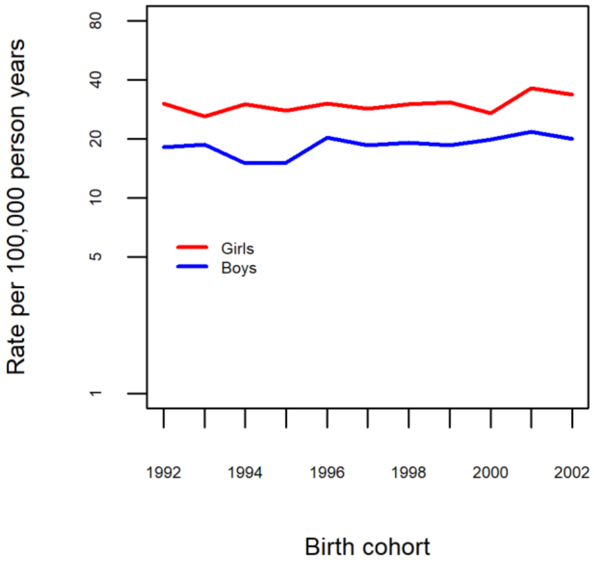

Figure S1: Sex-specific incidence rates per 100,000 PY of JIA in Danish birth cohorts from 1992–2002

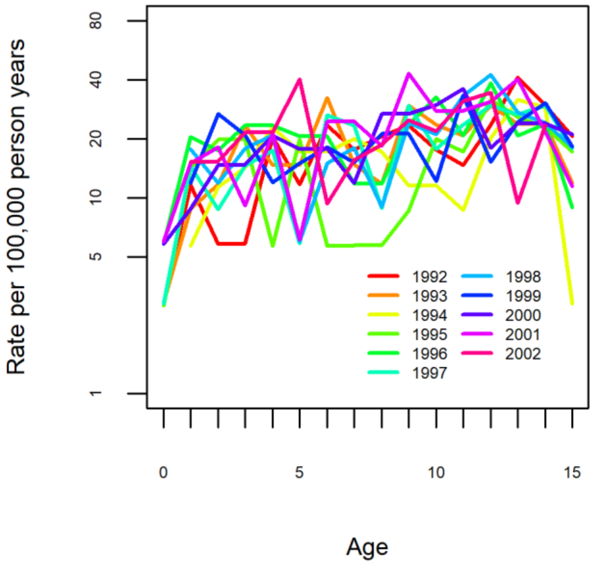

Figure S2: Birth-cohort specific incidence rates per 100,000 PY of JIA by age group for boys

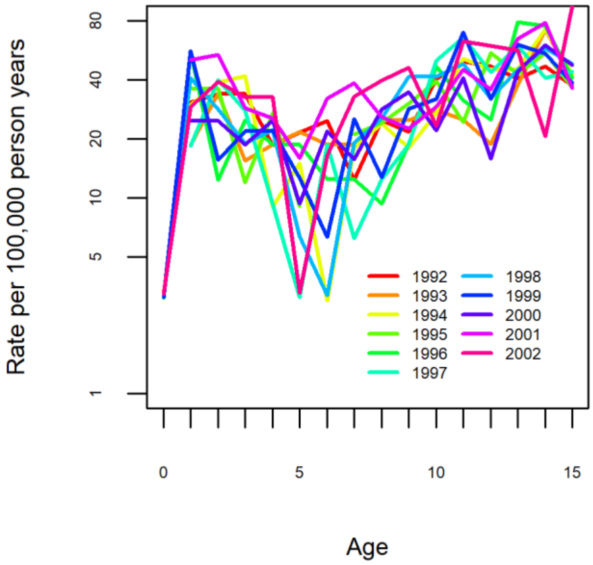

Figure S3: Birth-cohort specific incidence rates per 100,000 PY of JIA by age group for girls

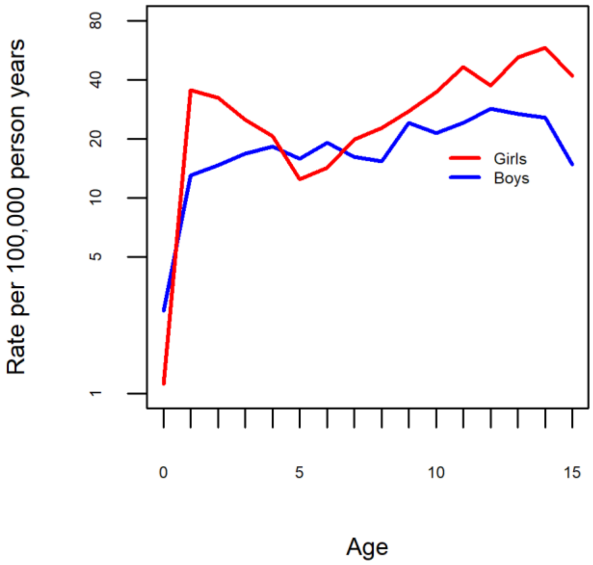

Figure S4: Total incidence rate of JIA by age group for boys and girls
